# Global profile of tRNA-derived small RNAs in gastric cancer patient plasma and identification of tRF-33-P4R8YP9LON4VDP as a new tumor suppressor

**DOI:** 10.7150/ijms.53220

**Published:** 2021-02-04

**Authors:** Yijing Shen, Xiuchong Yu, Yao Ruan, Zhe Li, Yaoyao Xie, Zhilong Yan, Junming Guo

**Affiliations:** 1 Department of Biochemistry and Molecular Biology, and Zhejiang Key Laboratory of Pathophysiology, School of Medicine, Ningbo University, Ningbo 315211, China.; 2 Ningbo No. 1 Hospital Affiliated to Ningbo University School of Medicine.

**Keywords:** tRNA-derived small RNAs (tsRNAs), tRNA-derived fragment (tRF), gastric cancer, tRF-33-P4R8YP9LON4VDP, proliferation.

## Abstract

Transfer RNA (tRNA)-derived small RNAs (tsRNAs) have been found to play important roles in the occurrence and development of cancers. However, the tsRNA profile in gastric cancer is unknown. In this study, we aimed to identify the global tsRNA profile in plasma from gastric cancer patients and elucidate the role of tRF-33-P4R8YP9LON4VDP in gastric cancer. Differentially expressed tsRNAs in the plasma of gastric cancer patients and healthy controls were investigated using RNA sequencing. The expression levels of tRF-33-P4R8YP9LON4VDP in the plasma of gastric cancer patients, healthy controls and gastric cancer cell lines were first detected by quantitative reverse transcription-polymerase chain reaction. The effects of tRF-33-P4R8YP9LON4VDP overexpression or downregulation in gastric cancer cells on proliferation, migration, apoptosis, and cell cycle were analyzed using the Cell Counting Kit‐8, scratch assay, Transwell assay, and flow cytometry, respectively. There were 21 upregulated and 46 downregulated tsRNAs found in plasma from gastric cancer patients. The significantly upregulated tsRNAs included tRF-18-S3M83004, tRF-31-PNR8YP9LON4VD, tRF-19-3L7L73JD, tRF-33-P4R8YP9LON4VDP, tRF-31-PER8YP9LON4VD, tRF-18-MBQ4NKDJ, and tRF-31-PIR8YP9LON4VD. The significantly downregulated tsRNAs included tRF-41-YDLBRY73W0K5KKOVD, tRF-18-07QSNHD2, tRF-28-86J8WPMN1E0J, tRF-29-86V8WPMN1EJ3, tRF-31-6978WPRLXN4VE, tRF-30-MIF91SS2P46I, tRF-26-MI7O3B1NR8E, tRF-30-RRJ89O9NF5W8, tRF-26-XIP2801MK8E, and tRF-35-V0J8O9YEKPRS93, *In vitro* studies showed that tRF-33-P4R8YP9LON4VDP inhibited proliferation of gastric cancer cells. In conclusion, tsRNAs such as tRF-33-P4R8YP9LON4VDP could serve as a novel diagnostic biomarker and target for gastric cancer therapeutics.

## 1. Introduction

Cancer is one of the most significant public health issues worldwide. Gastric cancer is a major cause of cancer-related death and accounts for the second highest mortality rate in the world 1, 2. Global cancer statistics in 2018 showed that gastric cancer was associated with 1/12 deaths 3. In China, according to the newly released annual report on cancer, gastric cancer ranks third for both cancer incidence and mortality, with 41 cases of gastric cancer per 100,000 elderly people 4. More than 80% of patients with early gastric cancer have no specific symptoms. Most gastric cancer patients are mid- to late-stage by the time of diagnosis. The 5-year survival rate for early gastric cancer patients can be greater than 90% if treated early 5,6. Currently, a highly sensitive and specific peripheral blood tumor biomarker for gastric cancer does not exist.

tRNA-derived small RNAs (tsRNAs) are a new type of non-coding small RNA derived from tRNAs 7,8. It has been reported that tsRNAs are widely found in various prokaryotes and eukaryotes, ranging from *E. coli* to mammals 9. The first discovered tsRNA was a small fragment derived from tRNA, also known as tRNA halves (tiRNAs), that appear in stress-induced situations 10. tiRNAs are small RNA fragment in the range of 31-40 nt produced by the cleavage of the anticodon loop of mature tRNAs and are specifically cleaved by ribonuclease or angiogenin (ANG) 11,12. Although this type of tsRNA were first referred to as stress fragments, subsequent studies have found that tiRNAs can also occur in non-stress situations 13,14. The other main type of tsRNAs are the tRNA-derived fragments (tRFs), which can be classified according to the specific cleavage position (such as tRF-5s, tRF-3s, and tRF-1s) 15,16. Both tRF-5s and tRF-3s are derived from mature tRNAs 17. tRF-5s are cleaved by ANG or Dicer and derived from the D loop to the 5'-end of mature tRNAs. tRF-3s are generated from the TψC loop to the 3'-end of mature tRNAs by cleavage with RNase Z or RNase P. tRF-1s, also known as 3' U‐tRFs, are derived from the 3'-end of the precursor tRNA transcript by cleavage with RNase Z 18,19. More recently, tRFs have attracted increasing attention because of the similarities with microRNAs (miRNAs) 20. For example, several tRFs can interact in an miRNA-like manner to utilize complementary sequences on target RNAs and regulate the expression of these target genes 7, 21-22. tsRNAs have been shown to regulate expression of cancer-associated genes at different levels by binding to Piwi and Argonaute proteins 23. Interestingly, a recent study has also identified tsRNAs as a novel potential tumor marker 24.

To date, multiple tsRNA databases have been established. For example, the first established tRFdb database contains tsRNA sequences for multiple species (including human) 25. The MINTbase database can be used to query specific data about the maximum abundance of tsRNAs and modification of its parent tRNAs 26. However, there are no standardized naming systems by now. Same tsRNAs even have different names in different databases. The makers of the tDRmapper database propose assigning a three-component name to each tsRNA. The first part represents the source of the parental tRNA of each tsRNA. The second part represents the size of the tsRNA, and the third part represents the regions where the tsRNA is located in the mature tRNAs or precursor RNAs 27.

In this study, we aimed to screen tsRNAs associated with gastric cancer and to explore the possible functions of tsRNAs in gastric cancer. We first analyzed the differential global profiles of tsRNAs between plasma samples from preoperative gastric cancer patients and healthy controls through small RNA sequencing. By comprehensively comparing data such as fold change and *P* values, tRF-33-P4R8yP9LON4VDP was selected for further study. The length of this tsRNA is 33 nt and it is numbered P4R8YP9LON4VDP in the MINTbase database. The expression level of tRF-33-P4R8YP9LON4VDP, which is one type of tRF, was measured in a larger cohort of plasma samples of gastric cancer patients and a control group. A Cell Counting Kit‐8 (CCK-8) assay, colony-formation assay, scratch assay, Transwell assay and flow cytometry were used to analyze the roles of tRF-33-P4R8YP9LON4VDP in gastric cancer cells.

## 2. Materials and methods

### 2.1. Clinical specimens and data collection

In this study, 89 fasting peripheral blood samples from gastric cancer patients collected one day prior to and seven days post-surgery, and 98 fasting peripheral blood samples from healthy donors were collected from Ningbo No. 1 Hospital Affiliated with Ningbo University School of Medicine from January 2017 to June 2018. All blood samples were stored in an ethylenediamine tetraacetic acid (EDTA)-coated vacutainer tube (BD Biosciences, Franklin Lakes, NJ, USA). After collection, the samples were centrifuged immediately in a centrifuge and plasma was collected and stored in a -80°C freezer for subsequent experiments 28.

All biopsy specimens were confirmed by pathological diagnosis. The 8^th^ tumor-node-metastasis staging system of the International Union Against Cancer in 2017 was used 29. All patients were diagnosed with primary gastric carcinoma and did not receive any preoperative radiotherapy, chemotherapy, targeted therapy, or immunotherapy. The Human Research Ethics Committee of Ningbo University approved all aspects of this study in accordance with the Declaration of Helsinki (No. 2019022501). Written informed consent was obtained.

### 2.2. RNA extraction, quality assessment, pretreatment, and sequencing

Total RNA was extracted and isolated from plasma samples using TRIzol LS reagent (Invitrogen, Carlsbad, CA, USA) according to the manufacturer's instructions. The concentration and purity of total RNA were determined using a NanoDrop™ Nd-1000 spectrophotometer (NanoDrop, Thermo Fisher Scientific, Inc., Wilmington, DE, USA). To eliminate redundant modifications that could interfere with the small RNA sequencing library preparation in the subsequent steps, total RNA was pretreated with the rtStar^TM^ tRF and tiRNA Pretreatment Kit (cat. no. AS-FS-005, Arraystar, Rockville, MD, USA) in accordance with the manufacturer's instructions. The construction and sequencing of the small RNA library was completed by Shanghai Kangcheng Company (Shanghai, China).

The sequencing reads were aligned with several small RNA databases, including those for ribosomal RNAs, small nuclear RNAs, small nucleolar RNAs, Piwi-interacting RNAs and miRNAs. The main intersected databases were the tRF database (http://genome.bioch.virginia.edu/trfdb/) and Mintbase (https://cm.jefferson.edu/MINTbase/). The expression levels of tsRNAs were normalized to a millionth of the total aligned tRNA reads and measured. The fold changes (i.e. the ratio of the group means) and the *P* values were mainly used to compare the characteristic changes between gastric cancer patients and the healthy control group. The tsRNAs with fold changes ≥2.0 and *P*<0.05 were considered to be significantly differently expressed. Volcano plots and heat maps were used to show differently expressed tsRNAs between the two groups.

### 2.3. Validation with quantitative reverse transcription-polymerase chain reaction

Quantitative reverse transcription-polymerase chain reaction (qRT-PCR) is the gold standard for quantifying gene expression levels 30. Total RNA extracted from the plasma samples of 89 pairs of gastric cancer patients and 98 healthy controls was pretreated to remove excess modifications using the rtStar^TM^ tRF and tiRNA Pretreatment Kit according to the manufacturer's instructions (Arraystar). First-strand cDNA was then produced using the rtStar™ First-strand cDNA Synthesis Kit (Arraystar). In short, 1 µl of the 3′ adaptor and 0.5 µl of RNA was spiked into 2 μg of pretreated RNA for a total volume of 8.8 µl in RNase-free water. The reaction was then incubated at 70°C for 2 min, followed by addition of 7.2 µl of 3' ligation reaction buffer and a subsequent incubation at 25°C for 1 h. Then, 1 µl 3' ligation enzyme mix and 1 µl RNase inhibitor was added and the reaction was incubated at the following temperatures: 75°C/5 min, 37°C/15 min, and 25°C/15 min. The 5' adaptor ligation system was then added and the reaction was incubated at 25°C for 1 h. Lastly, 8 µl of the first-strand synthesis reaction buffer, 3 µl 0.1 M DTT, 2 µl 2.5 mM dNTP mix, 1 µl RNase inhibitor, and 1 µl reverse transcriptase was added and the reaction was incubated at 45°C/1 h, then 70°C/15 min. The qRT-PCR reaction was performed on the Mx3005P Real-Time PCR System (Stratagene, Palo Alto, CA, USA) following the instructions of the GoTaqqPCR Master Mix Kit (Promega, Madison, WI, USA). Primers are shown in Supplemental Table 1 and were validated by sequencing the PCR products (Supplemental Fig. 1). The Δ*C*_t_ values were used to analyze the relative expression levels of tRF-33-P4R8YP9LON4VDP. Data were normalized to the expression of the endogenous reference gene, the U6 small RNA 31,32. All experimental data were obtained from three independent experiments.

### 2.4. Cell culture and transfection

The normal human gastric epithelial cell line GES-1 and gastric cancer cell lines, SGC-7901, AGS, and BGC-823, were purchased from the Chinese Academy of Medical Sciences Cancer Hospital (Beijing, China) and the Cell Bank of the Type Culture Collection of the Chinese Academy of Sciences (Shanghai, China). Roswell Park Memorial Institute (RPMI) 1640 Medium (HyClone, Los Angeles, CA, USA) was used to culture GES-1, SGC-7901, and BGC-823 cells. High Glucose Dulbecco's Modified Eagle's Medium (DMEM) (HyClone) was used to culture AGS cells. During experiments, both types of media contained 1% penicillin/streptomycin (Life Technologies, Carlsbad, CA, USA) and 10% fetal bovine serum (FBS) (Gibco, Grand Island, NY, USA). In addition, culturing of all cell lines was performed at 37°C and 5% CO_2_.

During transfection, cells at the logarithmic growth stage were first trypsinized and then seeded uniformly into a six-well plate. Cells were incubated with complete culture medium for about 24 h until cell confluency reached 60-70%, and then fresh complete culture medium was used to rinse the plate. Then 5 µl negative control (NC) mimics or inhibitor, tRF-33-P4R8YP9LON4VDP mimics or tRF-33-P4R8YP9LON4VDP inhibitor (GenePharma, Shanghai, China), and 5 µl Invitrogen™ Lipofectamine 2000 (Life Technologies) were mixed with 200 µl Opti-MEM I Reduced Serum Medium (Invitrogen, Carlsbad, CA, USA) and added to a six-well plate. The plate was then incubated with serum-free medium. The medium was then replaced with complete medium after a 4-6 h incubation. All cell function experiments were performed 24-48 h after transfection. Primer sequences of the NC mimics and inhibitor, and tRF-33-P4R8YP9LON4VDP mimics and inhibitor are shown in Supplemental Table 1.

### 2.5. Cell proliferation and colony formation assays

The CCK‐8 and colony formation assays were used to measure changes in cell proliferation. For CCK-8, transfected cells were first selected, trypsinized and seeded in 96-well plates (Corning Inc. Corning, NY, USA) at 5,000 cells per well. Cell activity was measured at 24 h, 48 h, 72 h, and 96 h after cell seeding, according to the manufacturer's instructions for the CCK‐8 assay (Dojindo, Tokyo, Japan). In brief, 10 µl CCK-8 reagent was added to each well and incubated for 4 h in the dark at 37°C and 5% CO_2_. The absorbance at 450 nm was then measured using The SpectraMax M5 Multi-Mode Microplate Reader (Molecular Devices, Silicon Valley, CA, USA).

For the colony formation assay, transfected cells were trypsinized and seeded into a 6-well plate at 1,000 cells per well. Cells were then cultured in complete medium at 37°C and 5% CO_2_ atmosphere. Two weeks later, 4% paraformaldehyde was used for fixation; and 0.1% crystal violet solution was used for staining. Finally, the chromogenic colonies were counted.

### 2.6. Migration assays

The changes in cell migration were measured using the scratch assay and Transwell assay. For the cell scratch assay, vertical lines in each well of a six-well plate were drawn using a 200 µl pipette tip. After rinsing, cells were observed with a CKCG3 microscope (Olympus, Tokyo, Japan), and three random fields were imaged. Cells were cultured in serum-free medium at 37°C and 5% CO_2_ for 48 h and photographed again. The distance of cell migration was measured using Image J Software Inc. (Rawak Software Inc, Stuttgart, Germany).

For the Transwell assay, transfected cells were first trypsinized with 0.25% trypsin-EDTA, followed by resuspension in serum-free medium. The resuspended cells were then seeded at 80,000 cells per well into precoated Matrigel (BD Bioscience) chambers (Corning). The upper chamber was inserted into a 24-well plate containing complete medium with 15% FBS. After incubation for 24 h at 37°C and 5% CO_2_, the cells remaining in the upper chamber were gently wiped off with a cotton swab. To fix the cells, chamber was immersed in 4% paraformaldehyde for 30 mins at room temperature. Cells were then stained with 0.1% crystal violet solution. Three fields were randomly selected with an inverted fluorescence microscope to count cells.

### 2.7. Cell apoptosis and cell cycle assays

For cell apoptosis analysis with the Cell Apoptosis Kit (Multisciences, Hangzhou, China), the transfected cells were first digested with EDTA-free trypsin and rinsed twice with phosphate buffered saline (PBS). Cells were then resuspended with 500 µl Binding Buffer (Multisciences), followed by addition of 10 µl propidium iodide and 5 µl Annexin V-FITC (Multisciences) in the dark for 5 min at room temperature. Cell apoptosis was assessed by flow cytometry (BD Bioscience). To detect changes in cell cycle distribution, cells were serum-starved for 24 h prior to transfection. Cells were then trypsinized and rinsed twice with PBS. Then 1 mL of DNA staining solution and 10 µl permeabilization reagent were added to the cells and the cells were incubated at room temperature for 30 min in the dark. Cell cycle changes were also measured by flow cytometry.

### 2.8. Data analysis

All experiments were repeated three times. SPSS V.19.0 Software (IBM, Almont, NY, USA) and GraphPad Prism 6 Software (GraphPad Software Inc, San Diego, CA, USA) were used for statistical analysis of experimental data. All data are represented by mean ± SD. In addition, Photoshop (Adobe, San Jose, CA, USA) and Image J software (Rawak Software Inc) were also used to process the data. The differences between the treated and control groups were analyzed and compared using a student's *t*-test. A χ^2^ test was used to evaluate the association between tRF-33-P4R8YP9LON4VDP expression levels and clinicopathological data of gastric cancer patients. For all analyses, a *P* value <0.05 for the two-tailed test was considered statistically significant.

## 3. Results

### 3.1. Small RNA sequencing results

**Figure 1:**
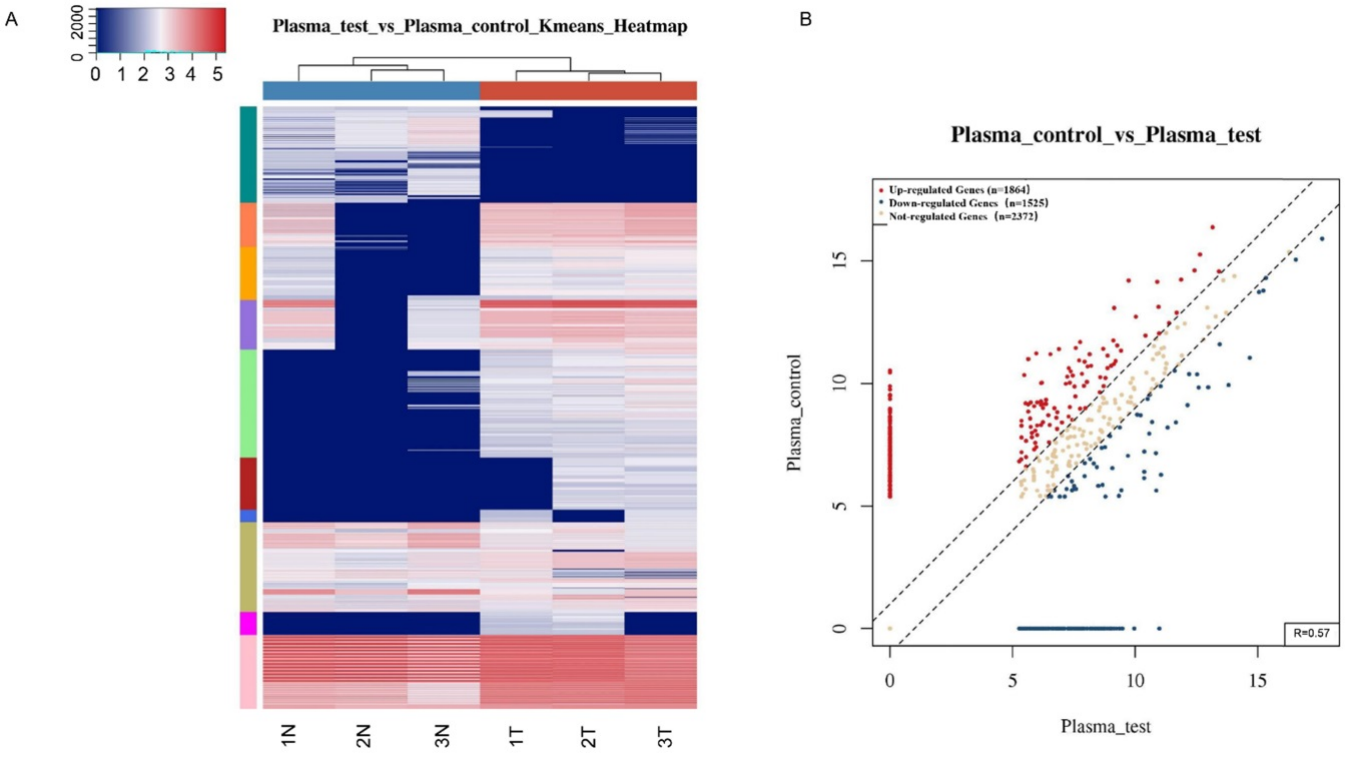
Differentially expressed tsRNAs between gastric cancer patient plasma and healthy donors. **(A)** Hierarchical clustering heatmap. Each row represents a tsRNA and each column represents a sample. T, cancer patient; N, healthy control. **(B)** Scatter plots between two groups. The counts per million values of all tsRNAs are plotted.

To identify tsRNAs associated with gastric cancer, small RNA sequencing was performed. Among the differentially expressed tsRNAs in plasma samples from the three gastric cancer patients and three healthy individuals (OED205624 https://www.biosino.org/node/) (Fig. 1), 67 tsRNAs were found to be significantly different (Supplemental Table 2; Supplemental Table 3). The upregulated tsRNAs include: tRF-18-S3M83004, tRF-31-PNR8YP9LON4VD, tRF-19-3L7L73JD, tRF-33-P4R8YP9LON4VDP, tRF-31-PER8YP9LON4VD, tRF-18-MBQ4NKDJ, and tRF-31-PIR8YP9LON4VD (Supplemental Table 2). The downregulated tsRNAs include: tRF-29-86V8WPMN1EJ3, tRF-31-6978WPRLXN4VE, tRF-35-PSQP4PW3FJIKE7, tRF-22-8BWS72092, tRF-25-PNR8YP9LON, tRF-29-79MP9P9NH525, tRF-19-V29K9U2Y, tRF-17-HR0VX6J, tRF-30-87R8WP9N1EWJ, tRF-20-9MVKS4I7, tRF-27-WJ9X0UD394N, tRF-20-V29K9UV3, tRF-27-87R8WP9N1E5, tRF-26-86J8WPMN1EE, tRF-30-MIF91SS2P46I, tRF-26-MI7O3B1NR8E, tRF-30-RRJ89O9NF5W8, tRF-26-XIP2801MK8E, tRF-35-V0J8O9YEKPRS93, tRF-41-YDLBRY73W0K5KKOVD, tRF-18-07QSNHD2, and tRF-28-86J8WPMN1E0J (Supplemental Table 3). Based on differences in fold change and the synchronous changes in tsRNAs between cancer patient plasma and healthy control plasma, tRF-33-P4R8YP9LON4VDP was used for further study.

### 3.2. Detection of tRF-33-P4R8YP9LON4VDP levels in gastric cancer patient plasma and gastric cancer cells

**Figure 2:**
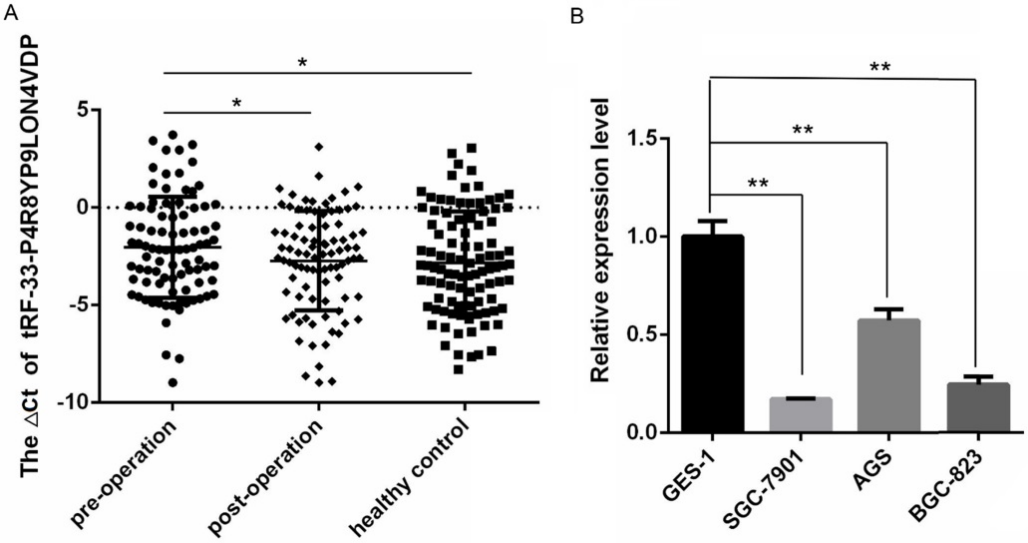
Differentially expressed levels of tRF-33-P4R8YP9LON4VDP in gastric cancer and healthy controls. (**A**) The levels of tRF-33-P4R8YP9LON4VDP in preoperative samples (*n*=89) were lower than those in postoperative (*n*=89) and healthy samples (*n*=98). Higher ΔCt values indicate low expression. *P<0.05. (**B**) The levels of tRF-33-P4R8YP9LON4VDP in the normal gastric epithelial cell line GES-1 were higher than those in gastric cancer cell lines, SGC-7901, AGS, and BGC-823. The relative expression was calculated using the 2^-ΔΔCt^ method.* n*=3, **P<0.01.

To validate the small RNA sequencing results, qRT-PCR was used to measure the expression of tRF-33-P4R8YP9LON4VDP in plasma samples from 89 gastric cancer patients one day prior to and seven days after surgery, and 98 healthy plasma samples. The expression of tRF-33-P4R8YP9LON4VDP in plasma samples of gastric cancer patients one day prior to surgery was significantly lower than that of healthy controls (*P*=0.0402). tRF-33-P4R8YP9LON4VDP levels were reduced in preoperative plasma samples of gastric cancer patients (*P*=0.0446) compared to postoperative plasma samples from gastric cancer patients (Fig. 2A).

The expression of tRF-33-P4R8YP9LON4VDP in the normal gastric epithelial cell line GES-1 and gastric cancer cell lines, SGC-7901, AGS and BGC-823, was also detected. The expression levels of tRF-33-P4R8YP9LON4VDP were significantly lower in three gastric cancer cell lines compared to the normal gastric epithelial cell lines (Fig. 2B), which was consistent with the trend in expression of tRF-33-P4R8YP9LON4VDP in plasma samples.

### 3.3. tRF-33-P4R8YP9LON4VDP inhibited the proliferation, migration, and apoptosis of gastric cancer cells *in vitro*

**Figure 3:**
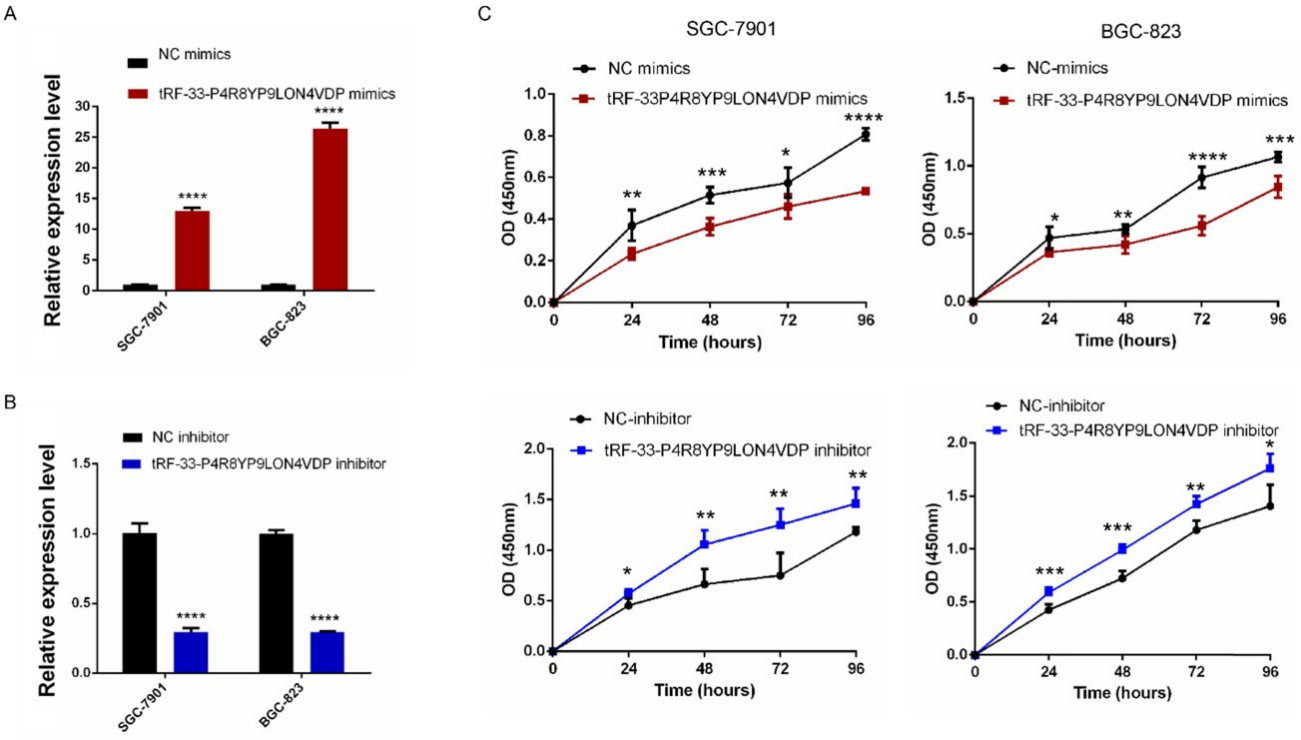
Proliferation of gastric cancer cells after tRF-33-P4R8YP9LON4VDP upregulation and downregulation. **(A and B)** Effect of upregulating and downregulating tRF-33-P4R8YP9LON4VDP in gastric cancer cells using tRF-33-P4R8YP9LON4VDP mimics or inhibitor. Relative expression was calculated using the 2^-ΔΔCt^ method. **(C)** Growth curves of gastric cancer cells after upregulating and downregulating tRF-33-P4R8YP9LON4VDP. NC, negative control. *n*=3, *P<0.05, **P<0.01, ***P<0.001, ****P<0.0001.

**Figure 4:**
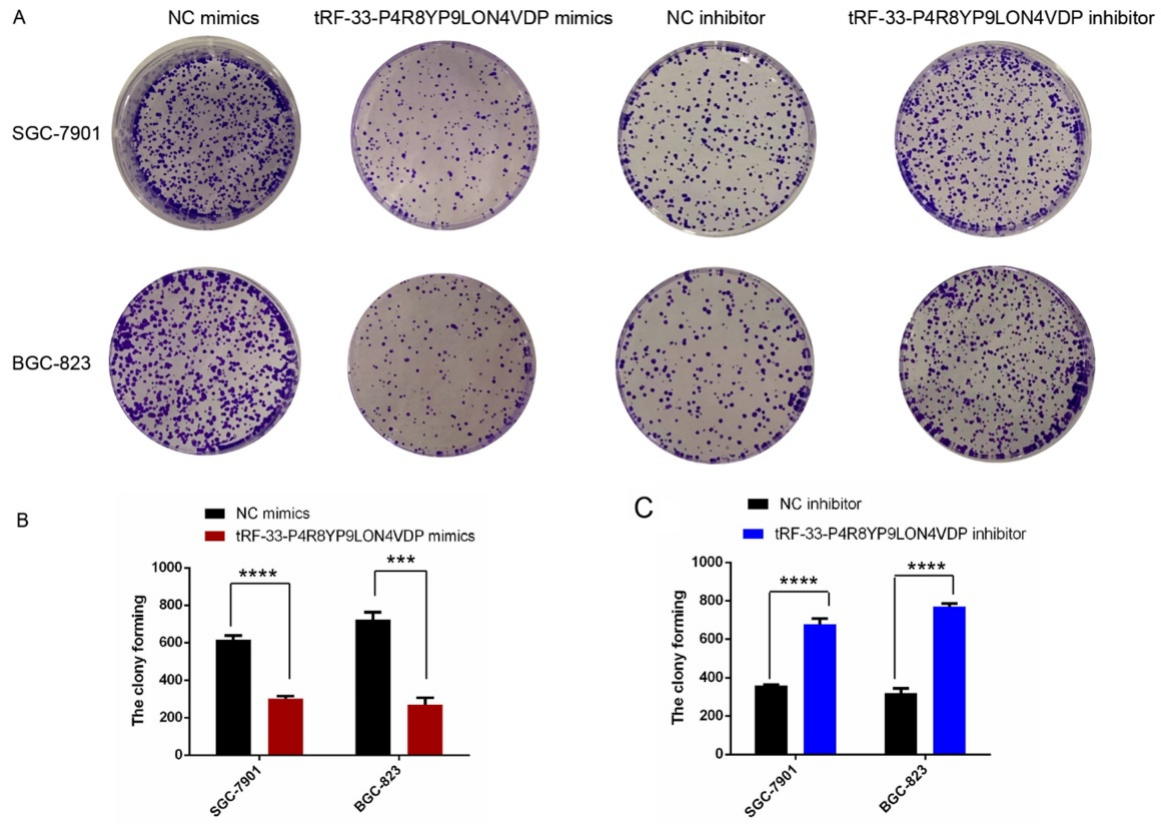
Colony formation of gastric cancer cells after tRF-33-P4R8YP9LON4VDP upregulation and downregulation. (**A**) Representative colony formation assays of SGC-7901 and BGC-823. (**B and C**) Quantitation from three colony formation experiments. NC, negative control. ***P<0.001, ****P<0.0001.

**Figure 5:**
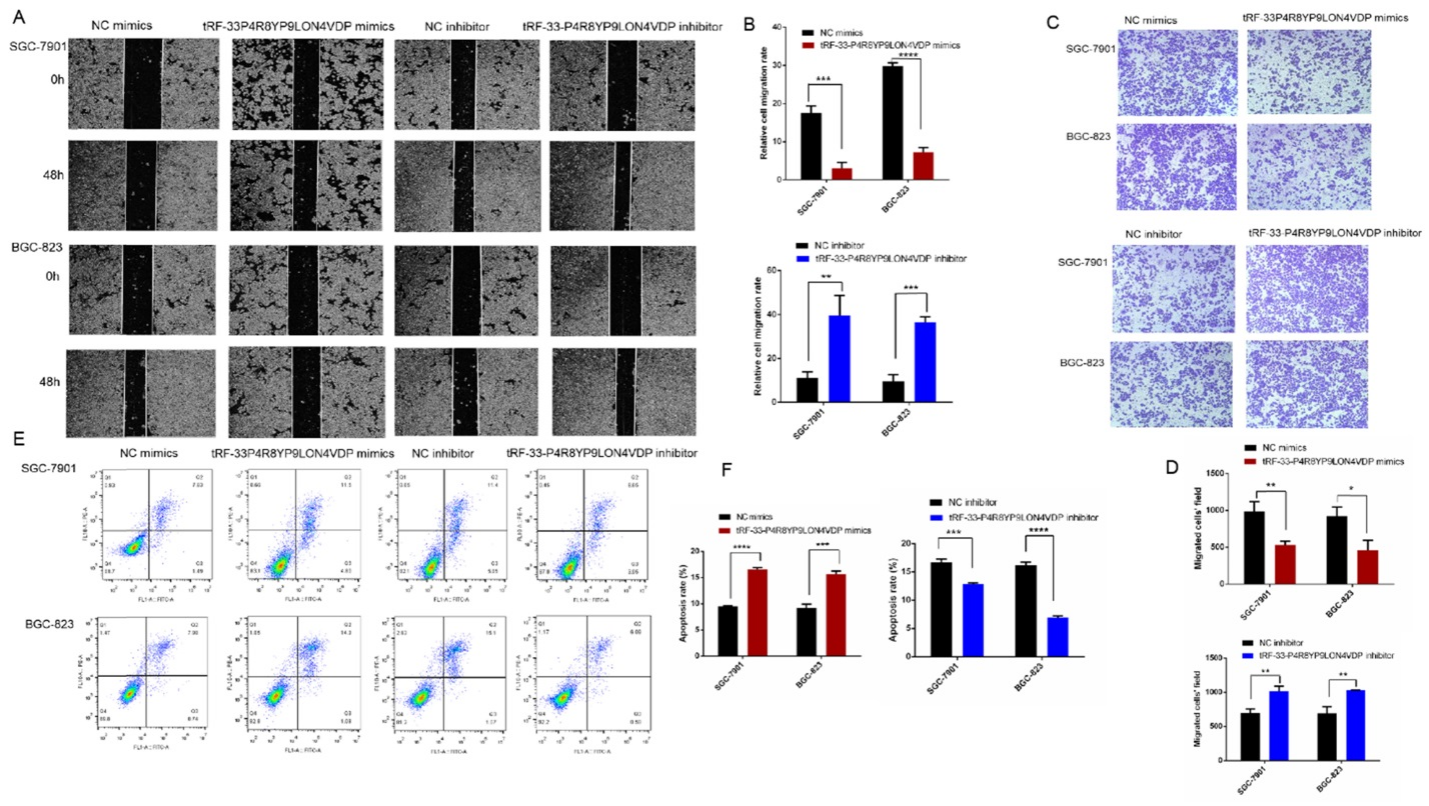
Effects of altering tRF-33-P4R8YP9LON4VDP expression with mimics or inhibitors on gastric cancer cell migration ability (**A-D**) and apoptosis (**E and F**). **(A)** Representative images of scratch assay experiments. **(B)** Quantitation of scratch assays. **(C)** Representative images of the Transwell assay. **(D)** Quantification of the Transwell assay. **(E)** Representative flow cytometry results of apoptosis. **(F)** Quantitation of three apoptosis experiments of apoptosis. NC, negative control. *P<0.05, **P<0.01, ***P<0.001, ****P<0.0001.

To further investigate the potential roles of tRF-33-P4R8YP9LON4VDP in gastric cancer, we upregulated and downregulated tRF-33-P4R8YP9LON4VDP using tRF-33-P4R8YP9LON4VDP mimics and tRF-33-P4R8YP9LON4VDP inhibitor, respectively, in SGC-7901 and BGC-823 cell lines. qRT-PCR results verified the efficiency of the upregulation and downregulation of tRF-33-P4R8YP9LON4VDP (Fig. 3A, B). We then evaluated the effect of tRF-33-P4R8YP9LON4VDP on gastric cancer cell proliferation. Compared with the control group, the proliferation of SGC-7901 and BGC-823 cells transfected with tRF-33-P4R8YP9LON4VDP mimics was inhibited, while proliferation was increased in SGC-7901 and BGC-823 cells after transfection with the tRF-33-P4R8YP9LON4VDP inhibitor, as assessed by the CCK-8 assay (Fig. 3C). Colony formation experiments further verified that the proliferation of gastric cancer cells was inhibited by transfection with tRF-33-P4R8YP9LON4VDP mimics, while proliferation was promoted by transfection with tRF-33-P4R8YP9LON4VDP inhibitors (Fig. 4). In addition, the cell scratch and Transwell migration assays were used to examine the changes in migration ability of gastric cells after altering tRF-33-P4R8YP9LON4VDP expression. The results of both experiments were consistent. Compared with the control group, transfection with tRF-33-P4R8YP9LON4VDP mimics decreased cell migration ability, while transfection with the inhibitor increased cell migration (Fig. 5A-D).

**Figure 6:**
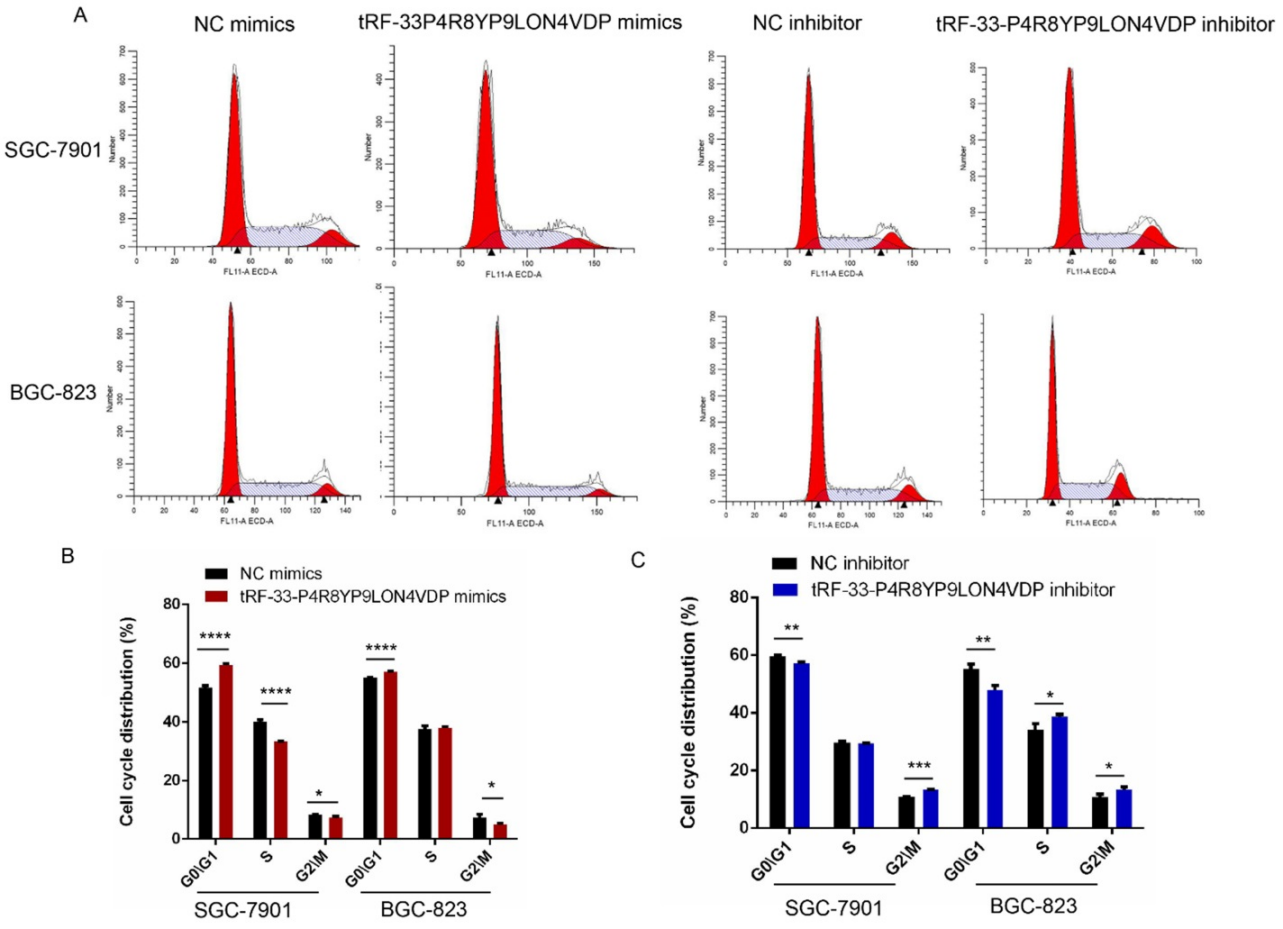
Cell cycle changes in gastric cancer cells after tRF-33-P4R8YP9LON4VDP upregulation and downregulation. (**A**) Representative flow cytometry results. (**B and C**) Quantitation of three experiments. NC, negative control. *P<0.05, **P<0.01, ***P<0.001, ****P<0.0001.

We also used flow cytometry to evaluate whether tRF-33-P4R8YP9LON4VDP affects gastric cancer cell apoptosis and cell cycle. Apoptosis assays revealed that tRF-33-P4R8YP9LON4VDP induced apoptosis in gastric cancer cells (*P*<0.05) (Fig. 5E, F). In addition, upregulation of tRF-33-P4R8YP9LON4VDP caused more cells to be distributed in G_1_ phase, suggesting that tRF-33-P4R8YP9LON4VDP arrests cells at G_0_/G_1_ (Fig. 6). We further found that cells were arrested at G_2_/M after transfection with a tRF-33-P4R8YP9LON4VDP inhibitor. These data indicate that tRF-33-P4R8YP9LON4VDP inhibits gastric cancer cells phenotypes.

## 4. Discussion

The incidence rate of cancer has been increasing steadily since the turn of the 21^st^ century. Worldwide, there are more than 14 million new cancer cases each year 33. In China, on average more than 10,000 people are diagnosed with cancer every day and 7.5 people are diagnosed with cancer every minute 33. In recent decades, researchers have made great strides in cancer research and broadening the understanding of cancer. Studies have shown that an increasing number of ncRNAs are associated with tumors 34-38. For example, long non-coding RNAs (lncRNAs) can affect the development and progression of tumors 34, 35. Circular RNA can also affect many diseases, including cancers 36-38. However, until recent years, researchers have discovered that the sequence and size of tsRNAs have many previously-overlooked biological functions 39, 40. For example, tsRNAs can be used as modifiers to influence protein translation and affect the cellular response to stress 39-41. In addition, tsRNAs can also affect cell migration, proliferation, or invasion, which influences the occurrence and development of diseases such as cancers 42, 43. There are some similarities in length between tsRNAs and miRNAs. Studies have found that tRF-3s derived from tRNA^Leu^ can weaken protein translation in non-small cell lung cancer cells, like miRNAs 44. The sequences of miR-1247a and miR-1247b are consistent with tRFs derived from tRNA^Lys3^ and tRNA^Lys5^ through database comparison 45. However, the relationship between other non-coding small RNAs and tsRNAs remains to be explored. There is increasing evidence that tsRNAs are key regulators of cancer-related processes and could serve as novel diagnostic biomarkers and therapeutic targets for tumor therapy in the future 46, 47.

Gastric cancer is a complex biological process involving many molecular abnormalities. Genes and ncRNAs, such as miRNAs and lncRNAs, have been shown to be associated with gastric cancer 48, 49. However, little is known about the role of tsRNAs in gastric cancer. This study is the first comprehensive evaluation of tsRNA profiles in gastric cancer (Fig. 1). A total of 67 differentially expressed tsRNAs were identified between plasma samples from gastric cancer patients and healthy controls (Supplemental Table 2, Supplemental Table 3). These results provide fundamental information to further the understanding of the roles of tsRNAs in the occurrence and development of gastric cancer, and to help in the search for diagnostic biomarkers and therapeutic targets.

In this study, tRF-33-P4R8YP9LON4VDP was selected as a promising target tsRNA. Compared with plasma samples from gastric cancer patients seven days after surgery and healthy controls, qRT-PCR results showed low levels of tRF-33-P4R8YP9LON4VDP in plasma samples from gastric cancer patients one day before surgery (Fig. 2A). Interestingly the expression level of tRF-33-p4R8yP9LON4VDP in the expanded cohort of samples (89 gastric cancer patients and 98 healthy controls) was different compared to that obtained by small RNA sequencing in the three paired samples (Supplemental Table 2). This discrepancy could be because of the small sample size used in the sequencing experiment 50. Such an observation suggests that RNA microarray profiling or a sequencing screen in which a small number of samples are used, an expanded number of samples tested with RT-qPCR should be used to confirm the results 49.

We have shown that tRF-33-P4R8YP9LON4VDP inhibited proliferation and migration, promoted apoptosis and altered the cell cycle of gastric cancer cell lines BGC-823 and SGC-7901 (Figs. 3-6). These results were also consistent with our findings that tRF-33-P4R8YP9LON4VDP was lowly expressed in gastric cancer plasma and cell lines (Fig. 2). Therefore, it is speculated that tRF-33-P4R8YP9LON4VDP may play a suppressive role in gastric cancer. In other tumors such as ovarian cancer, tRF^leu-CAG^ has been observed to regulate downstream target genes 42. The expression of *Aurka* was inhibited when tRF^Leu-CAG^ was downregulated 42. However, it is still unclear whether tRF^Leu-CAG^ can directly affect *Aurka* 42. In high-grade serous ovarian cancer, tRF-03357 was found to downregulate the expression of HMBOX1, which belongs to the hepatocyte nuclear factor family 42. HMBOX1 has also been reported to be involved in the occurrence of a variety of tumors. It has been found that HMBOX1 is upregulated in gastric cancer and leads to a poor prognosis by promoting cell proliferation and migration 51. It is possible that tRF-33-P4R8YP9LON4VDP can also inhibit gastric cancer by targeting downstream genes. Additional research on tsRNAs could lead to enhanced understanding of how tsRNAs could be utilized in cancer therapeutics.

In conclusion, the findings provided in this study of gastric cancer-associated tsRNAs could provide new insights for novel types of diagnostic biomarkers and therapeutic targets of gastric cancer. The tsRNA tRF-33-P4R8YP9LON4VDP, as a representative gastric cancer-associated tsRNA, may play a tumor suppressor role in gastric cancer and could serve as a potential therapeutic target of gastric cancer.

## Supplementary Material

Supplementary figures and tables.
