# Supplementary material for: Global profile of tRNA-derived small RNAs in gastric cancer patient plasma and identification of tRF-33-P4R8YP9LON4VDP as a new tumor suppressor
Source: Int J Med Sci. 2021 Feb 4;18(7):1570–9. doi: 10.7150/ijms.53220 (PMC7976566; doi:10.7150/ijms.53220)
Supplement: Supplementary file 1 — Supplementary figures and tables. [file ijmsv18p1570s1.pdf]

Supplemental tables and figures

Supplemental Table 1. The sequences of primers

Name	Sequence
tRF-33-P4R8YP9LON4VDP	F:5' GCATGGGTGGTTCAGTGGTA3'
	R:5' GTGCTCTTCCGATCTCAGGC3'
U6	F:5' GCTTCGGCAGCACATATACTAAAAT 3'
	R:5’ CGCTTCACGAATTTGCGTGTCAT 3’
NC mimics	Sense:5' UUCUCCGAACGUGUCACGUTT3'
	Antisense:5' ACGUGACACGUUCGGAGAATT3'
NC inhibitor	5' CAGUACUUUUGUGUAGUACAA3'
tRF-33-P4R8YP9LON4VDP mimics	Sense:5'GCAUGGGUGGUUCAGUGGUAGAAUUCUCGCCUG 3'
	Antisense:5' GGCGAGAAUUCUACCACUGAACCACCCAUGCUU3'
tRF-33-P4R8YP9LON4VDP inhibitor	5' CAGGCGAGAAUUCUACCACUGAACCACCCAUGC3'

Supplemental Table 2. Upregulated expressed tsRNAs between plasmas from gastric cancer patients and healthy controls

tsRNA Name	Mintbase ID	tRNA	Sequence (5'→ 3')	Fold change	*P* value
AS-001051	tRF-18-S3M83004	tRNA_Ser_AGA/TGA	GTAGTCGTGGCCGAGTGG	2.54	0.0268
AS-007217	/	tRNA_Gly_GCC/CCC	GCATTGTGGTTCAGTGGTAGAATT CTCGCC	2.73	0.0010
AS-007238	/	tRNA_Gly_GCC/CCC	GCATTGGTGGTTCAGTGGTAGAAT TCTCGCT	2.83	0.0238
AS-000011	tRF-31-PNR8YP9LON4VD	tRNA_Gly_GCC/CCC	GCATTGGTGGTTCAGTGGTAGAAT TCTCGCC	3.30	0.0080
AS-009402	/	tRNA_His_GTG	CCGTGATCGTATAGTGGTTAGTAC TCTGT	4.79	0.0413
AS-007371	/	tRNA_Gly_CCC	GCGTTGGTGGTTCAGTGGTAGAAT TCTCGCC	6.29	0.0142
AS-010960	/	tRNA_Glu_TTC	GCGTTGTGGTTCAGTGGTAGAATT CTCGCC	126.13	<0.0001
AS-005386	tRF-19-3L7L73JD	tRNA_Val_AAC	CCGTAGTGTAGTGGTTATC	158.60	0.0049
AS-010967	/	tRNA_Gly_GCC	GCATAGGTGGTTCAGTGGTAGAA TTCTCGCT	163.17	0.0018
AS-009209	/	tRNA_Ser_AGA/TGA	GTAGTCGTGGCCGAGTGA	164.57	0.0017
AS-001290	tRF-33-P4R8YP9LON4VDP	tRNA_Gly_GCC	GCATGGGTGGTTCAGTGGTAGAA TTCTCGCCTG	169.07	0.0004

AS-009370	/	tRNA_Gly_CCC	GCATTGGTGGTTTAGTGGTAGAATTCTCGCC	195.13	0.0025
AS-010959	/	tRNA_Glu_TTC	GCATCGTGGTTCAGTGGTAGAATTCTCGCC	197.64	0.0153
AS-008129	/	tRNA_Val_CAC	GCATGGTGGTTCAGTGGTAGAATTCTCGCC	203.18	0.0054
AS-001263	tRF-31-PER8YP9LON4VD	tRNA_Glu_TTC	GCAATGGTGGTTCAGTGGTAGAATTCTCGCC	230.71	0.0030
AS-008249	/	tRNA_Met_CAT	CGGAAGCGTGCTGGGCCT	239.33	<0.0001
AS-007440	/	tRNA_Gly_GCC	GCATTGGTGGTTCAGTGGTAGAATTCTTGCC	241.83	0.0013
AS-007757	/	tRNA_Gly_GCC	GCATAGGTGGTTCAGTGGTAGAATTCTCGCC	260.08	0.0024
AS-008085	/	tRNA_Val_CAC	GCACTGGTGGTTCAGTGGTAGAATTCTCGCT	323.91	0.0002
AS-004537	tRF-18-MBQ4NKDJ	tRNA_Met_CAT	CGGAAGCGTGCTGGGCCC	517.72	0.0002
AS-001443	tRF-31-PIR8YP9LON4VD	tRNA_Thr_CGT	GCACTGGTGGTTCAGTGGTAGAATTCTCGCC	569.40	0.0033

Supplemental Table 3. Upregulated expressed tsRNAs between plasmas from gastric cancer patients and healthy controls

tsRNA Name	Mintbase ID	tRNA	Sequence (5'→ 3')	Fold change	*P* value
AS-00060	tRF-29-86V8WPMN1EJ3	tRNA_Glu_TTC	TCCCATATGGTCTAGCGGTTAG GATTCCT	2.153	0.0493
AS-001399	tRF-31-6978WPRLXN4VE	tRNA_Pro_TGG/CGG/AGG	GGCTCGTTGGTCTAGGGGTATG ATTCTCGCT	2.29	0.0052
AS-000056	/	tRNA_Glu_TTC	TCCCACATGGTCTAGCGGTTAG GATTCCTGG	3.23	0.0434
AS-008719	/	tRNA_Pro_TGG/CGG/AGG	GGCTCGTTGGTCTAGGGGTATG ATTCTCGCC	3.40	0.0089
AS-001363	tRF-35-PSQP4PW3FJIKE7	tRNA_Lys_CTT	GCCCGGCTAGCTCAGTCGGTA GAGCATGGGACTCT	3.56	0.0196
AS-006813	tRF-22-8BWS72092	tRNA_Gln_CTG	TCAAATCTCGGTGGAACCTCCA	4.04	0.0353
AS-008211	/	tRNA_Glu_CTC	TCCCTGGTGGTCTAGTGGTTAG GATTCGGT	4.52	0.0121
AS-000688	tRF-25-PNR8YP9LON	tRNA_Gly_CCC/GCC	GCATTGGTGGTTCAGTGGTAGA ATT	4.56	0.0389
AS-001138	tRF-29-79MP9P9NH525	tRNA_Val_AAC	GTTTCCGTAGTGTAGTGGTTAT CACGTTC	4.92	0.0370

AS-008225	/	tRNA_Glu_CTC	TCCCTGTGGTCTAGTGGTTAGG ATTCGGC	5.16	0.0170
AS-000507	tRF-19-V29K9U2Y	tRNA_Gln_TTG	TAGGATGGGGTGTGATAGG	5.69	0.0484
AS-005996	tRF-17-HR0VX6J	tRNA_Leu_AAG/TAG	ATCCCACCGCTGCCACC	5.79	0.0380
AS-008549	/	tRNA_Val_CAC	GCTTCTGTAGTGTAGTGGTTAT CACGTTCGCCTCACACGCG	5.96	0.0025
AS-000546	tRF-30-87R8WP9N1EWJ	tRNA_Glu_CTC	TCCCTGGTGGTCTAGTGGTTAG GATTCGGC	6.15	0.0111
AS-009217	/	tRNA_Val_CAC	GTTTCCGTAGTGTAGTGGTTAT CACGTTCGCCTGACACGCGAA AGGTCCCC	6.33	0.0143
AS-009379	/	tRNA_Val_CAC	GTTTCCGTAGTGTAGTGGTTAT CACGTTCGCCTCACACGCGAA AGGTCCCC	7.00	0.0280
AS-009381	/	tRNA_Val_CAC	GCTTCTGTAGTGTAGTGGTTAT CACGTTCGCCTCACACGCGA	7.32	0.0087

AS-007517	/	tRNA_Gln_CTG	GGTTCCATGGTGTAATGGTTAGCACTCTGGACTCTGAATCCAGCGATCCGA	7.75	0.0182
AS-004874	tRF-20-9MVKS4I7	tRNA_Pro_TGG/CGG/AGG	TGGTCTAGGGGTATGATTCT	7.96	0.0487
AS-001757	tRF-27-WJ9X0UD394N	tRNA_Asn_GTT	TCGGCTGTTAACCGAAAGGTTGGTGGT	8.68	0.0084
AS-009271	/	tRNA_Gly_GCC	GCATTGGTGGTTCAGTGGTAGAATTCTCGCCTGCCACGCGGGAGGCCCGGG	9.24	0.0043
AS-009295	/	tRNA_Gly_GCC	GCATTGTGGTTCAGTGGTAGAATTCTCGCCTGCCACGCGGGAGGCCCGGGT	9.48	0.0172
AS-000508	tRF-20-V29K9UV3	tRNA_Gln_TTG	TAGGATGGGGTGTGATAGGT	10.13	0.0130
AS-009255	/	tRNA_Glu_TTC	TCCCACATGGTCTAGCGGTTAGGATTCT	14.74	0.0003
AS-008205	/	tRNA_Gly_CCC	CATTCTTGCGACCCGGGTTCGTTTCCCGGGCGGCGCACCA	15.36	0.0461

AS-000543	tRF-27-87R8WP9N1E5	tRNA_Glu_CTC	TCCCTGGTGGTCTAGTGGTTAG GATTC	15.74	0.0097
AS-009320	/	tRNA_Val_CAC	GCTTCTGTAGTGTAGTGGTTAT CACGTTCGCCTCACACGCGAA AGGTCCCC	25.20	0.0004
AS-000581	tRF-26-86J8WPMN1EE	tRNA_Glu_TTC	TCCCACATGGTCTAGCGGTTAG GATT	29.33	0.0016
AS-007204	/	tRNA_Val_CAC	GCTTCTGTAGTGTAGTGGTTAT CACGTTCGCT	144.03	0.0040
AS-004019	tRF-30-MIF91SS2P46I	tRNA_Lys_CTT	CGGCTAGCTCAGTCGGTAGAG CATGGGACT	153.09	0.0048
AS-001746	tRF-26-MI7O3B1NR8E	tRNA_Asn_GTT	CGGCTGTTAACCGAAAGGTTG GTGGT	156.20	0.0241
AS-000531	tRF-30-RRJ89O9NF5W8	tRNA_Gln_TTG	GGTCCCATGGTGTAATGGTTAG CACTCTGG	158.08	0.0141
AS-001967	tRF-26-XIP2801MK8E	tRNA_Cys_GCA	TGACTGCAGATCAAGAGGTCC CCGGT	160.22	0.0116

AS-004102	tRF-35-V0J8O9YEKPRS93	tRNA_Lys_CTT	TAGAGCATGGGACTCTTAATCCCAGGGTCGTGGGT	176.19	0.0058
AS-009212	/	tRNA_Glu_TTC	TCCCATATGGTCTAGCGGTTAGGATTCT	202.82	0.0181
AS-008562	/	tRNA_Gly_TCC	GCGTTTGTGGTATAGTGGTGAGCATAGCTGCCTTCCAAGCAGTTGACCCGG	222.12	0.0372
AS-000197	tRF-41-YDLBRY73W0K5KKOVD	tRNA_Asn_GTT	TTAACCGAAAGGTTGGTGGTTCGAGCCCACCCAGGGACGCC	224.49	0.0005
AS-008457	/	tRNA_Ile_TAT	CTCACCTGGAGCATGTTTTCT	241.66	0.0235
AS-007320	/	tRNA_Und_NNN	TCCCTGGTGGTCTAGTGGTTAGGATTCT	260.93	0.0279
AS-009197	/	tRNA_Ala_AGC	TCCCCGGCACCTCCACCAT	274.75	0.0266
AS-009198	/	tRNA_Pro_TGG	TTGGGGTGCGAGAGGTCCCGGGT	337.73	0.0385

AS-008686	/	tRNA_Val_CAC	GCTTCTGTAGTGTAGTGGTTAT CACGTTCGCCTCACACGCA	358.32	0.0407
AS-005818	tRF-18-07QSNHD2	tRNA_Ser_TGA	ACCCTGCTCGCTGCGCCA	368.50	0.0355
AS-008266	/	tRNA_Gly_TCC	GCGTTGGTGGTATAGTGGTTAG CATAGCTGCCTTCCAAGCAGTT GACCCGG	412.56	0.0144
AS-009273	/	tRNA_Pro_TGG/AGG	ATCCCGGACGAGCCCCCATT	458.65	0.0101
AS-000583	tRF-28-86J8WPMN1E0J	tRNA_Glu_TTC	TCCCACATGGTCTAGCGGTTAG GATTCC	1419.14	0.0139

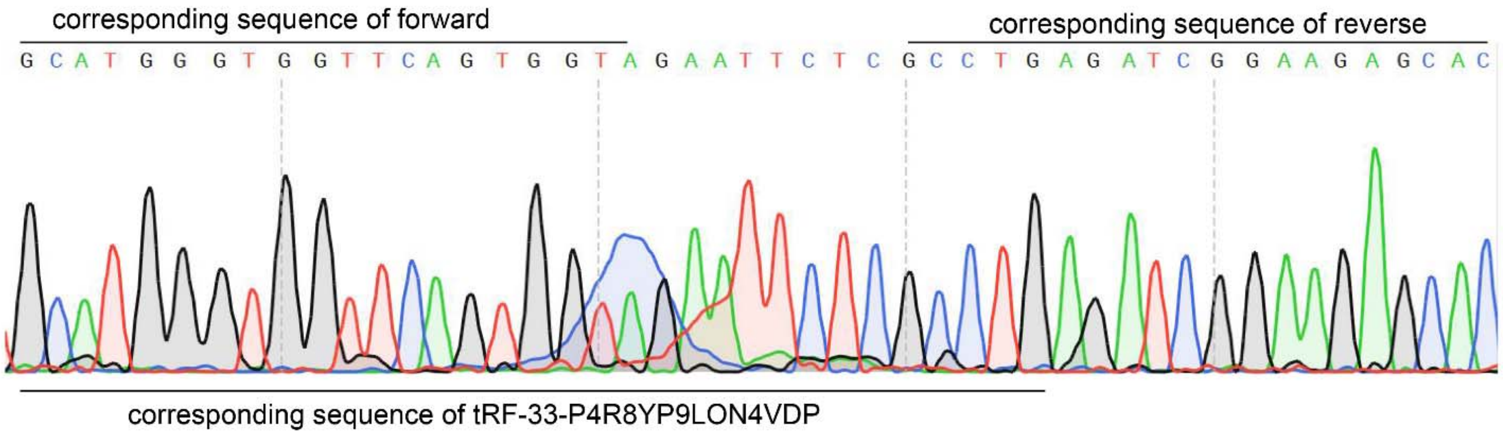

Supplemental Figure 1. Sequence results of qRT-PCR products of tRF-33-P4R8YP9LON4VDP.
